# A mindfulness and compassion-based program applied to pregnant women and their partners to decrease depression symptoms during pregnancy and postpartum: study protocol for a randomized controlled trial

**DOI:** 10.1186/s13063-019-3739-z

**Published:** 2019-11-28

**Authors:** Olga Sacristan-Martin, Miguel A. Santed, Javier Garcia-Campayo, Larissa G. Duncan, Nancy Bardacke, Carmen Fernandez-Alonso, Gloria Garcia-Sacristan, Diana Garcia-Sacristan, Alberto Barcelo-Soler, Jesus Montero-Marin

**Affiliations:** 1 Primary Care Prevention and Health Promotion Research Network (RedIAPP), Valladolid, Spain; 2 International School of Doctorate Studies, National University of Distance Education (UNED), Madrid, Spain; 3 Department of Personality Psychology, National University of Distance Education (UNED), Madrid, Spain; 4 Aragon Institute for Health Research (Instituto de Investigacion Sanitaria Aragón (IIS Aragon), Miguel Servet Hospital, Psychiatry Service, University of Zaragoza, Zaragoza, Spain; 5 Primary Care Prevention and Health Promotion Research Network (RedIAPP), Zaragoza, Spain; 6 Center for Child & Family Well-Being, School of Human Ecology, University of Wisconsin-Madison, Madison, WI USA; 7 Mindful Birthing and Parenting Foundation, Oakland, CA USA

**Keywords:** Perinatal and postpartum depression, Stress in pregnancy, Childbirth, Mindfulness, Compassion, RCT

## Abstract

**Background:**

Pregnancy and the postpartum period are times of great change for women and their partners, often bringing substantial challenges and stress. Approximately 10%–20% of women suffer from mood disorders such as depression in the perinatal period. There are risks involved in using psychopharmacological interventions to treat perinatal depression. Mindfulness and compassion-based educational programs could be efficacious and cost-effective options for the prevention and treatment of perinatal mood disorders. The aim of this study is to assess the efficacy of an adapted Mindfulness-Based Childbirth and Parenting (MBCP) program that includes compassion training for pregnant women in primary care (PC) settings in the Spanish National Health System to decrease perinatal depression.

**Methods:**

A multicenter randomized controlled trial (RCT) will be conducted. Participants will be pregnant women (*n* = 122) and their partners who wish to participate. They will be enrolled and assessed in PC settings and randomly assigned to either: (1) an adapted MBCP educational program tailored to the Spanish National Health System + treatment as usual (TAU); or (2) TAU only. The main outcome to be assessed will be depression, evaluated with the Edinburgh Postnatal Depression Scale (EPDS). Secondary outcomes will include self-reported measures of perceived stress, affects, mindfulness, self-compassion, maternal self-efficacy, and use of health and social services. Patients will be assessed at four timepoints: baseline; post-treatment; and at three and six months after childbirth. Intention-to-treat and per-protocol analyses will be carried out using linear regression mixed models. Effect sizes will be estimated using Cohen’s d.

**Discussion:**

Perinatal depression is a significant health problem. An effective and low-cost childbirth education program that incorporates mindfulness and compassion practices may be a beneficial preventive complementary healthcare modality for expectant women and their partners. This study will be the first multicenter RCT in Spanish PC settings using adapted MBCP and compassion practices to reduce symptoms of depression during pregnancy and the postpartum period.

**Trial registration:**

ClinicalTrials.gov, NCT03247491. Registered on 31 July 2017.

## Background

Perinatal depression (PD), which includes major and minor depressive episodes that occur during pregnancy and/or in the first 12 months after delivery, is one of the most common conditions that can develop during pregnancy and the postpartum period [1]. The prevalence of PD in developing countries is approximately 20%; in developed countries, it is in the range of 10%–15% [2]. Untreated PD can have devastating effects on women, infants, and their families [3–5], so much so that NICE guidelines in the UK recommend screening for PD in primary care (PC) settings [6].

Prenatal depression is one of the main risk factors for postpartum depression. It often goes undiagnosed and untreated, with serious consequences for the mother and, by extension, the infant, including growth delays in the developing fetus, prematurity, low birth weight, disorganized infant sleep patterns, and less responsiveness to the external environment [4]. Among Spanish women, the prevalence of prenatal depressive symptoms is approximately 15% [7]. In addition, prenatal depression appears to affect men; thus, the importance of the presence of the partner in interventions tailored to improve couple wellbeing during pregnancy and postpartum is encouraged [8, 9].

Other risk factors for postpartum depression include a young age (e.g. the prevalence of postpartum depression in teenage mothers is much higher than for adult mothers), a previous history of depression, and the presence of postpartum blues: a transient mood disorder characterized by mild depressive symptoms that is common in new mothers [10–13]. A large-scale study conducted in Spain [14] found rates of minor postpartum depression was in the range of 11%–17%, while major postpartum depression was in the range of 8%–11%. Given the prevalence of PD and the adverse effects of this disorder for women, children, and families [3–5], the development and implementation of cost-effective programs and interventions have important health implications. Usual treatments for PD include counseling, psychotherapy, and antidepressant medication. However, there is evidence of risks to both the fetus and breastfeeding infants that limits the use of antidepressants [12, 15], and antidepressant exposure during pregnancy may increase susceptibility to disorders such as hypertension for the expectant woman [16].

Mindfulness-based programs (MBPs) are educational mind–body courses that have the specific purpose of training the mind through meditation practice to adopt a non-judgmental awareness focused on the present moment [17]. In addition, compassion is a particular orientation of the mind that recognizes the universality of suffering in the human experience and cultivates the capacity to meet that suffering with kindness and empathy [18]. It is characterized by the presence of sensitivity to suffering and a commitment to prevent and alleviate it with equanimity and patience [19]. Evidence is growing that compassion is an important mechanism in MBPs and some researchers advocate explicit compassion training within MBPs [20].

MBPs have shown to be beneficial for those with symptoms of depression and other mental disorders [21, 22]. Moreover, some evidence suggests that learning and practicing mindfulness skills during pregnancy may improve both a mother’s symptoms of depression and a baby’s weight at birth [23]. Incorporating mindfulness and compassion into childbirth education could offer pregnant women and their partners at risk for PD, or currently experiencing depression, an alternative strategy for addressing this mood disorder without the stigma that can be associated with psychotherapy or counseling and the risks of antidepressant medication for the mother and the baby [24]. It may also offer a preventive strategy accessible to all pregnant women, as PD can arise without previous risk factors [12].

In addition, mindfulness training has been used as a tool for coping with both chronic and acute pain [25–28]. Thus, a mindfulness and compassion-based program could be useful for expectant women for coping with pain and discomforts often encountered during pregnancy, for childbirth-related pain as well as pain that can arise in the postpartum period, including during breastfeeding. This approach could provide an innovative and complementary skills-based educational approach that promotes physical and mental health and wellbeing during pregnancy, childbirth, and the postpartum period. Such a program could also be beneficial for pregnant women suffering from PD who prefer to avoid medications that may have adverse effects on the fetus and themselves [29], for women who wish to be as thoroughly prepared as possible for whatever may arise during childbirth [30], and for women who have a disposition for making positive behavior changes to improve their physical/mental health during pregnancy [31].

Programs such as Mindful Motherhood [32], Mindfulness-Based Childbirth Education (MBCE) [33], MindBabyBody [34], and the Mindfulness-Based Childbirth and Parenting (MBCP) program [35] have adapted mindfulness training for pregnancy, childbirth, and the postpartum period. MBCP, from which the protocol used in the present study has been specifically adapted, has been shown to decrease fear of childbirth [36] and led to important maternal mental health benefits including improvements in childbirth related appraisals and prevention of postpartum depression symptoms [24]. Other programs, which have been adapted from Mindfulness-Based Cognitive Therapy (MBCT) [37–39], have targeted pregnant women suffering from anxiety and depression and have yielded promising results. In addition, researchers in compassion-focused therapy (CFT) have created compassion interventions for prevention and treatment of PD [40, 41].

The Spanish National Health System (NHS) provides free universal healthcare regardless of financial condition or nationality. However, most regions in Spain do not have specific healthcare professionals who take care of women’s perinatal mental health. With approximately 400,000 births per year in Spain [42], cost-effective and accessible interventions for those coping with PD—as well as a strategy for prevention—are greatly needed. Within this context, the primary aim of the present study will be to compare the effectiveness of an adapted MBCP program with compassion practices that includes treatment as usual (TAU) tailored for delivery in the Spanish NHS and implemented in the second trimester, with an active control group receiving TAU only, to assess possible improvements in depressive symptomatology in pregnant women.

## Methods

### Study design

This is a multicenter randomized controlled trial (RCT) with two parallel groups: (1) adapted MBCP educational course with compassion practices delivered for pregnant women and their partners + TAU; and (2) TAU only, which consists of a childbirth education course provided by PC midwives. This protocol has followed the SPIRIT guidelines [43] Additional file 1. The trial registration number of the study is ClinicalTrials.gov NCT03247491.

### Setting and study sample

Participants will be pregnant women in the second trimester of pregnancy living in the city of Valladolid (Spain) who are served by the Spanish NHS. Partners of the expectant women will be encouraged to participate in the courses. Participants considered for inclusion will be: (1) women in weeks 6–25 of pregnancy; (2) able to read, write, and understand Spanish; (3) age ≥ 18 years ; and (4) have signed a written informed consent document following an informed consent procedure. Exclusion criteria will include: (1) any diagnosis of disease that may affect the central nervous system, such as brain pathology or traumatic brain injury; (2) other psychiatric diagnosis or acute psychiatric illness, such as substance dependence or abuse, a history of schizophrenia or other psychotic or eating disorders; (3) any medical, infectious, or degenerative disease that may affect mood; (4) presence of delusional ideas, hallucinations, or at risk for suicide; and (5) currently under psychopharmacological medication or under psychopharmacological treatment.

### Sample size

The sample size estimation was based on the expectation of a moderate standardized mean difference between groups on depressive symptoms at post-birth of *d* = 0.59. Like the protocol that will be used in the present study, this effect size was found in a recent RCT that used a modified MBCP program [24] compared to a TAU active standard childbirth preparation course with no mind–body components. This effect size is also similar to that obtained in other studies using other programs [44], a pilot study evaluating the effectiveness of MBCE [33], a cohort study assessing the MindBabyBody program [34], and a brief pilot adaptation based upon the MBCP program [9]. Considering a statistical power of 80%, a 5% significance level in a between-group interaction with a local alpha of 0.017 in the first test—using Benjamini–Hochberg’s procedure—and a dropout rate in the range of 15%–20%, as has been observed in these types of studies [45], 61 participants are needed in each group, for a total sample size of 122 women.

### Recruitment

**Fig. 1:**
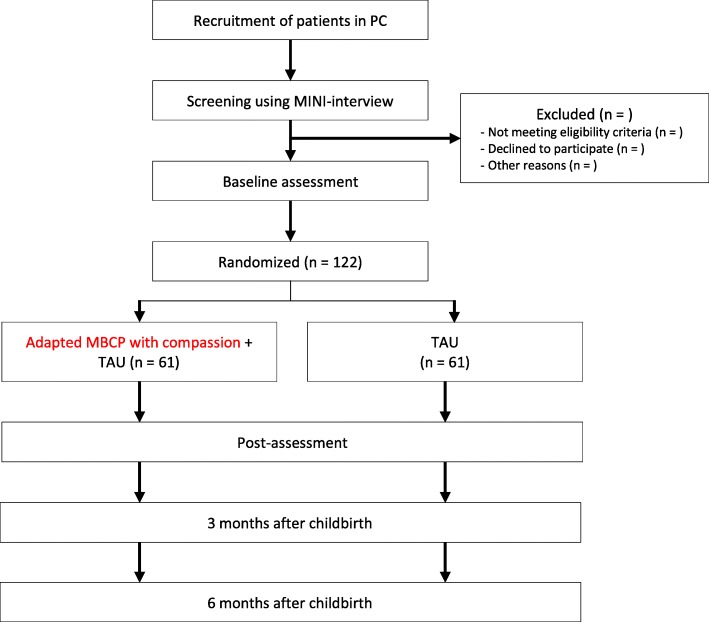
Study flowchart

**Fig. 2:**
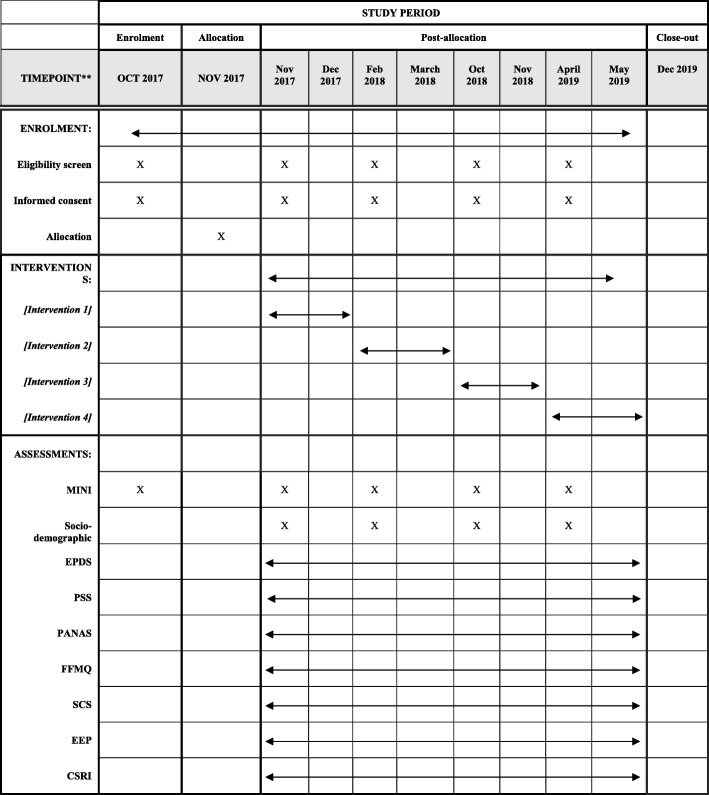
Schedule of enrolment, interventions, and assessment

Participants will be recruited from 11 PC urban healthcare centers in the city of Valladolid, Spain through referrals from midwives and obstetricians. When a health provider identifies a pregnant woman who might be a potential participant, they will facilitate contact with the primary study researcher who will arrange a meeting with both the pregnant woman and her partner. During this interview, the study characteristics will be explained, including the main objectives, potential benefits and adverse events, an explanation regarding the assigned home meditation practice, and the option to end their participation in the study at any time. Partners will be encouraged to participate in all of the sessions and the assigned home practices. If a pregnant woman is interested in taking part in the study, the researcher will give her an information booklet with additional details describing the trial. Within three days after having signed the written informed consent form, participants will be interviewed by an independent researcher who will administer the MINI International Neuropsychiatric Interview [46] in order to assess eligibility related to the inclusion and exclusion criteria. If the pregnant woman fulfills all of the study criteria, the same researcher will administer the baseline tests. An independent researcher will conduct the randomization procedure after the baseline assessment. Recruitment will be done consecutively to complete the sample size over an expected 24-month period. Flowcharts giving an overview of the study design and the study timeline are summarized in Figs. 1 and 2, respectively.

### Randomization, allocation, and masking of study groups

Women who want to participate in the trial who fulfill the study criteria will be assigned consecutive numbers and will be allocated to one of the two study groups using a simple – not stratified – random number sequence using a computer program generated by a member of the research team who does not know the study aims. The allocation will be carried out by a researcher of the “Primary Care Prevention and Health Promotion Research Network” (REDIAPP) who is not involved in the study. The number sequence will be concealed until intervention groups are assigned. Thus, participants will have to agree to participate in the study before the randomization process and will not be informed of their group allocation until after completion of the pre-treatment measures. Because of the nature of the intervention, participants cannot be blinded to their group allocation. Study personnel conducting psychological assessments will be masked to participants’ treatment conditions and will be specially advised not to ask for this information. The researcher who administers the baseline assessments will be unaware of which treatment group the patient will be assigned. This researcher will be different from the one who will facilitate the rest of study assessments by means of an online procedure. Midwives and obstetricians will be also unaware of the patients’ randomized assigned group for as long as possible. In addition, the statistician who will conduct the primary analysis of the data will be blind to which condition the patient is assigned.

### Interventions

All participants included in the study, whether they receive the adapted MBCP program with compassion practices or not, will be treated by their general practitioner (GP), obstetricians, and midwives according to TAU at a PC level. Women who belong to the same health center will receive the TAU from the same staff. The adapted MBCP program arm will receive a combined treatment, which consists of the MBCP program with compassion practices tailored to the Spanish NHS plus TAU. The program will take place during the second trimester of pregnancy, before participating in the TAU childbirth classes that are taught at their site during the third trimester.

#### Treatment as usual (TAU)

TAU consists of a childbirth education program that is offered at no charge to pregnant women and their partners by the local midwives in PC facilities in the Spanish NHS. Women usually attend this program in groups of 8–12 couples in the third trimester of pregnancy (during weeks 28–36). It covers basic topics about pregnancy, delivery, postpartum, breastfeeding, and care of a newborn baby. The duration of the TAU course is usually 16 h and includes both information-based classes and some instruction in basic relaxation and breathing techniques to cope with new stressful situations.

#### Adapted MBCP with compassion practices tailored to the Spanish NHS

The main objective of the MBCP program [47] is to teach mindfulness meditation for decreasing stress during pregnancy, reducing pain and fear during childbirth, to support parenting with wisdom and compassion, and to interrupt intergenerational patterns of suffering [48]. The course schedule is 3 h once a week for nine weeks, a 7-h silent practice day on the weekend between classes 6 and 7, and a reunion class after all the women have given birth. Although the MBCP program is designed for couples, pregnant women without a partner or whose partner cannot attend are also welcomed, as are pregnant women with other support people. A recommended class size is 8–12 couples. Various formal mindfulness meditation exercises are practiced in each class; participants are also asked to practice the meditations daily at home using audio recordings throughout the course. Formal mindfulness meditation and the attitudes cultivated in a mindfulness practice are fully integrated into the curriculum, which also includes instruction regarding the physiology and mind–body dimensions of pregnancy, labor, childbirth, breastfeeding, adjustment in the postpartum period, and attending to the needs of a newborn. Mind–body pain coping skills for childbirth and awareness skills for coping with stress in daily life are also taught. Other elements include encouraging a sense of connection or community among participants in order to minimize social isolation and its resulting negative effects on the mental health of new parents.

The intervention that will be used in this study is an adaptation of the basic MBCP program to: (1) include an explicit compassion component; and (2) be tailored to fit the Spanish population and the existing NHS programs for expectant women and their partners, improving adherence. It will be implemented at a PC level, with a duration of 10 sessions (eight sessions before childbirth and two sessions after childbirth, at three months and six months postpartum). Each class in this adapted MBCP program is 2 h long. Because all women receive free childbirth education classes from their midwives at their PC center, the adapted MBCP program will offer the mindfulness and compassion meditation practices from the original MBCP program without the childbirth information. In addition, this Spanish adaptation does not include the silent day of mindfulness practice as in the original MBCP program. While the value of this day of practice is clear, it is logistically difficult to implement it within the Spanish healthcare context as the PC facilities are closed during the weekends. However, the practices taught during the day of silence in the foundational MBCP program will be included in class 7. This class 7 has been adapted to cover all the practices offered during the course as well as the silent retreat day, including walking meditation and an interpersonal mindful speaking and listening inquiry between partners. There will be four adapted MBCP groups, with approximately 15 pregnant participants and their partners in each group. All of the classes will be held at same health center location. The total number of hours in the adapted MBCP program, including the TAU, will be 36.

During the adapted MBCP program with compassion exercises, participants will learn 14 formal mindfulness and compassion practices: awareness of breathing meditation; body scan meditation; compassionate body scan meditation; being with baby meditation; mindful yoga; walking meditation; creating a safe place meditation; loving-kindness meditation; sitting meditation; self-compassion meditation; coping with pain ice cube meditations; a three-step breathing meditation; compassionate touch meditation; and interpersonal mindful speaking and listening meditation. Participants will receive audio recordings of each of these practices to be used in their assigned daily home practices. All the participants will be asked to keep a daily diary of their home practice, as well as a calendar of pleasant and unpleasant events. See Appendix for a detailed description of all the elements of the adapted MBCP program.

### Instruments

All participants included in the trial will be assessed at pre-test/baseline, post- intervention, and at three and six months after childbirth (approximately six and 12 months after inclusion). The study instruments that will be used are summarized in Table 1.

**Table 1:** Study instruments

Variable	Area	Type	Time	Application
MINI	Psychiatric disorders	Nominal	Baseline	Researcher A (screening)
Sociodemographic	Age, marital status, education, occupation, nulliparity, and previous episodes of depression	Various	Baseline	Researcher A (baseline)
EPDS	Perinatal depression	Treated as interval	Baseline, post-treatment, and 3-month and 6-month follow-up	Researcher A (baseline) Online (follow-ups)
PSS	Perceived stress	Treated as interval	Baseline, post-treatment, and 3-month and 6-month follow-up	Researcher A (baseline) Online (follow-ups)
PANAS	Positive and negative affectivity	Treated as interval	Baseline, post-treatment, and 3-month and 6-month follow-up	Researcher A (baseline) Online (follow-ups)
FFMQ	Facets of mindfulness	Treated as interval	Baseline, post-treatment, and 3-month and 6-month follow-up	Researcher A (baseline) Online (follow-ups)
SCS	Facets of self-compassion	Treated as interval	Baseline, post-treatment, and 3-month and 6-month follow-up	Researcher A (baseline) Online (follow-ups)
EEP	Satisfaction and self-efficacy feelings about motherhood	Treated as interval	3-month and 6-month follow-up	Online (follow-ups)
CSRI	Use of health and social services	Treated as interval	Baseline and 6-month follow-up	Researcher A (baseline) Online (follow-up)

#### Main outcome

The primary outcome will be depressive symptom severity as assessed by the Edinburgh Postnatal Depression Scale (EPDS) [49]. The EPDS is a 10-item self-report scale used to assess the common symptoms of depression (e.g. “I have blamed myself unnecessarily when things went wrong”) during the perinatal period, both pre- and postnatally. It is one of the most widely used instruments to evaluate the severity of PD in clinical trials [50]. Each item of the EPDS is scored on a 4-point scale (from 0 to 3), with the total scale score in the range of 0–30. The validated Spanish version of the EPDS has a cutoff point of ≥ 11 to identify the presence of probable perinatal depression in women, obtaining an area under the curve of 0.98, with also good sensitivity and specificity values [51].

#### Secondary outcomes

A list of sociodemographic variables, such as age, marital status, education, occupation, nulliparity, and any previous depression episodes will be collected.

The Perceived Stress Scale (PSS) [52] is a widely used self-report instrument that evaluates the degree to which situations in one’s life are appraised as stressful. Items are designed to assess how unpredictable, uncontrollable, and overloaded respondents have found their lives to be during the last month. It consist of 14 items (e.g. “In the last month, how often have you found that you could not cope with all the things that you had to do?”) with a 5-point response scale from 0 (“never”) to 4 (“very often”) and a total score in the range of 0–56. Higher scores indicate greater perceived stress. The validated Spanish version of the PSS, which has demonstrated appropriate psychometrics, will be used [53].

The Positive and Negative Affect Schedule (PANAS) [54] consists of two 10-item scales that provide measures of positive affect (e.g. “interested”) and negative affect (e.g. “irritable”), with answers ranging in a Likert-type scale from 1 (“very slightly or not at all”) to 5 (“extremely or very much”). Participants are asked to rate the extent to which they have experienced each affective state, resulting in a total score in the range of 10–50. The Spanish version of the PANAS, adapted with adequate psychometrics and designed to assess affective states over the last week, will be used [55].

The Five Facet Mindfulness Questionnaire (FFMQ) [56] evaluates five facets of personal disposition towards being mindful in daily life situations. It is composed of 39 items to assess the subscales of observing (e.g. “When I’m walking, I deliberately notice the sensations of my body moving”), describing (e.g. “I’m good at finding words to describe my feelings”), acting with awareness (e.g. “When I do things, my mind wanders off and I’m easily distracted” – item reversed), non-judging of inner experience (e.g. “I make judgments about whether my thoughts are good or bad” – item reversed), and non-reactivity to inner experience (e.g. “I perceive my feelings and emotions without having to react to them”). The FFMQ is rated on a Likert-type scale, ranging between 1 and 5 points (from 1 = “never or very rarely true” to 5 = “very often or always true”). Total scores are in the range of 39–195, being that a high score indicates a high level of dispositional mindfulness. The validated Spanish version of the FFMQ that has demonstrated appropriate psychometric properties will be used [57].

The Self-Compassion Scale (SCS) [58] is a self-report measure of self-compassion. It consists of 26 items that assess how respondents perceive their actions toward themselves in times of difficulty, measuring facets of self-kindness (e.g. “I try to be loving towards myself when I’m feeling emotional pain”), common humanity (e.g. “I try to see my failures as part of the human condition”), and mindfulness (e.g. “When I’m feeling down, I tend to obsess and fixate on everything that is going wrong” – item reversed). Items range between 1 (“almost never”) to 5 (“almost always”). The SCS has shown appropriate psychometrics, and it allows for a unidimensional total score in the range of 26–130 [59]. The validated Spanish version of the SCS will be used [60].

The Parental Evaluation Scale (EEP) [61] is a self-administered measure to assess self-efficacy feelings about motherhood in women with children aged 0–2 years. It is composed of 10 items (e.g. “I feel like I do a good job as a mother”) that use a 10-point Likert-type scale from 0 (“Totally disagree”) to 10 (“Totally agree”). The EEP gives a global score of maternal self-efficacy in infants’ mothers in the range of 0–100, with higher values indicating greater self-efficacy. The validated Spanish version of the EEP has demonstrated appropriate psychometric characteristics [61].

The Client Service Receipt Inventory (CSRI) [62] is a questionnaire for gathering information about the use of healthcare and social services, as well as other economic variables (e.g. time of sickness absence, etc.). The version that will be used in this study has been designed to collect retrospective data on service utilization during the previous six months from baseline assessment and from the six-month follow-up measure. The CSRI-Spanish validated version, that has good psychometric properties, will be used [63].

### Analysis strategy

Results will be reported according to the CONSORT guideline statement [64]. Sociodemographic data at baseline will be presented by means of frequencies (percentages), medians (interquartile range), and means (SD), according to their level of measurement and statistical distribution. Visual inspection on the baseline data will be carried out to check the success of randomization through the two treatment conditions.

The primary efficacy analysis will use an intention-to-treat (ITT) base comparing the main outcome – EPDS – between arms in all the time points as a continuous variable. We will use a repeated measures (RM) design using linear mixed-effects regression models, including participants and the corresponding health center in the random part of the model, by means of the restricted maximum likelihood method (REML). Estimations of slope coefficients (and their 95% confidence intervals [CI]), adjusted for age, nulliparity, previous episodes of depression, and timing of receipt of intervention, will be calculated. To observe the specific trajectories through the study and to evaluate whether possible differences caused by the condition treatment are consistent over time, we will consider the “treatment × time” interaction. In parallel, a per-protocol analysis, with a minimum dose assumed to be ≥ 50% attendance [65], will also be carried out.

If missing data occur in the sample, multiple imputations by using chained equations to replace missing values will be calculated after ensuring data are missing at random (MAR), and as long as there are < 40% missing data in the corresponding variable to ensure validity of imputations [66]. Cohen’s *d* statistic, as an effect size (ES) measure of group differences will be estimated by means of pooled pre-test SDs to weight for the differences in the pre–post means [67]. Values of *d* = 0.20 are regarded as small, 0.50 as medium, and 0.80 large. Secondary analyses will comprise the PSS, PANAS, FFMQ, SCS, and EEP, and will use the same analytical strategy described above.

Cost-effectiveness and cost-utility procedures of analysis will also be conducted calculating incremental cost-effectiveness (ICER) and incremental cost-utility (ICUR) ratios. The effectiveness of the interventions will be estimated by means of the difference between the EPDS score at baseline and at follow-up; utility will be estimated using quality-adjusted life-year (QALYs) at follow-up. QALYs will be calculated using the area-under-the-curve (AUC). In addition, cost-utility planes will be plotted.

The overall alpha level will be set at 0.05 using two-sided tests and considering Benjamini–Hochberg’s correction for the primary outcome analyses as a way to balance between errors. However, no corrections will be made for secondary outcome analyses.

## Discussion

MBPs are effective alternative strategies for improving mental and physical health and wellbeing [65], for reducing symptoms of depression [22], for preventing depression relapse [68], and for managing pain and reducing stress [25, 26, 69]. They have also been proposed as potentially beneficial for expectant parents preparing for childbirth for managing pain during pregnancy and labor, reducing the risk of PD, and increasing the availability of parental attention for the infant [70]. With these last aims, several MBPs have specifically been adapted for this population [32–35, 37–39]; some of them have also included compassion techniques [40, 41].

No particular difficulties are expected in relation to recruitment of participants for the present study. However, since recruitment of participants will depend to a large extent on the attitudes of the PC providers who will be recommending the program to their patients, the study will be explained to them in detail before recruitment begins. It is expected that by providing this pre-recruitment information to PC physicians, possible negative attitudes about the study will be minimized.

Other potential difficulties may be that since patient participation in the study will be voluntary, there could be a higher rate of drop-out due to unforeseen life circumstances or conditions arising from the health of the mother or baby. Additionally, the only measures to be used in this study will be self-report questionnaires. Therefore, the data collected will have the limitations of this particular methodology.

### Clinical implications

To the best of our knowledge, this is the first study aiming to implement an adapted MBCP program which specifically adds a compassion component for preventing and treating PD in the Spanish healthcare context. If positive results are achieved, it could have a high impact on this important mental health issue that affects not only women but their partners and their newborn children [4, 5]. Moreover, if this intervention is cost-effective, it could be economically suitable for implementation in PC health centers throughout Spain. Currently, childbirth education classes in Spain are offered by midwives free of charge in all the PC health centers. If midwives were trained to teach this adapted MBCP program, pregnant women, their infants, and their partners might experience important and multiple benefits that come from learning mindfulness and compassion skills and practices for preventing PD.

## Trial status

The protocol version is 3 (25/09/2018). Recruitment began on June 2017 and will finish approximately on June 2019.

### Supplementary information

**Additional file 1.** SPIRIT 2013 Checklist: Recommended items to address in a clinical trial protocol and related documents.

## Data Availability

All study information will be confined in secure drawers with limited access. Electronic data files will be password-protected. Participant codes and personal information will be stored in a separate password-protected file. Only the researchers directly involved in the study will have access to the dataset. Paper-based data entry will be double-checked and possible out-of-range values will be revised. The study results will be presented via peer-review publications and congresses. The datasets used and/or analyzed during the current study will be available from the corresponding author on reasonable request.

## Appendix

**Table 2:** Elements of the adapted MBCP program

Class	Key concepts	Formal mindfulness practices	Interpersonal mindfulness practices	Formal home practices	Informal home practices
1	Participants learn how present moment awareness during labor and delivery can support the normal physiology of childbirth and may help develop a healthy, compassionate relationship between parent and child and between partners	Raisin meditation Awareness of Breathing meditation	Group sharing about personal and interpersonal changes in pregnancy provides couples the opportunity to normalize the stresses they may be experiencing and start creating a safe and nurturing environment among the participants	15-min Awareness of Breathing practice with audio recordings	Participants are encouraged to bring present moment awareness to routine activities of daily living, such as walking, taking a shower, driving, brushing their teeth, and preparing meals. This practice begins to set the foundation for a more responsive and less reactive parenting
2	Guided reflection about the motivation to participate in the intervention provides expectant parents an opportunity for sharing hopes and fears about pregnancy, childbirth, and parenting The Body Scan is introduced as a tool that helps participants to: • increase body awareness; • learn to be with sensations that are pleasant, unpleasant, and neutral; • experience the impermanence of physical sensations; • notice reactivity regarding thoughts and emotions in relation to physical sensations; • connect with the unborn baby; • begin to develop the skill of uncoupling the sensory component of pain from its emotional and cognitive components	Awareness of Breathing meditation Body Scan Being with Baby practice	Group sharing about guided reflection and group inquiry after Body Scan meditation promotes participant’s feelings of connection and common humanity	Continue Awareness of Breathing 5–10 min every day (with or without audio recordings). 30-min Body Scan with audio recordings	Continue Mindfulness of Routine Activities Practice of Being with Baby throughout the day, using the sensations from the baby’s movements as an opportunity and a reminder to come back to the body and the present moment
3	Participants learn - how fearful anticipations of pain may trigger a cascade of adverse stress reactions that can negatively affect the process of childbirth through the mind–body pathways of the neuroendocrine system - how mindfulness supports the normal physiology of labor with an attitude of acceptance and openness - the difference between primary and secondary suffering and how mindfulness helps participants be with the intense physical sensations of childbirth without adding the secondary suffering of reactivity - that moments *between* contractions can be experienced with equipoise and calm rather than with fear or worry about future pain or negative memories of past pain	Awareness of Breathing Meditation Compassionate Body Scan	Sharing in small groups of three or four about participant’s experiences practicing the Body Scan facilitates participants connecting at deeper levels as a community of practice Large group inquiry to share experiences practicing the Body Scan at home encourages participants to address and normalize common challenges	Continue Awareness of Breathing 5–10 min every day (with or without audio recordings) 30-min Body Scan with audio recordings	Continue Mindfulness of Routine Activities Continue Being with Baby practice Pleasant Events Calendar
4	Mindful Yoga adapted to pregnancy is introduced for the first time Noticing sensations of stretching and contracting and paying attention to the times of ease between poses prepares participants for noticing sensations of contracting and the moments of ease between contractions during childbirth Phrases from Loving-kindness practice during ice pain practice are also introduced. This practice may be especially useful for partners, who can sometimes experience empathic distress and feel helpless to alleviate their pregnant partner’s pain during labor	Sitting Meditation Yoga Practice Pain Practice with ice cubes to induce unpleasant sensations. A variety of mindfulness practices are offered, including - Breath awareness - Focusing attention directly on the unpleasant physical sensations - Moving awareness back and forth between sensations in the hand and the breath - Counting breaths - Turning up the corners of the mouth - Visualizing an image of a baby - Abiding in a safe place - Practicing a Body Scan between “ice sensations.” - Expanding awareness to the body as a whole, noticing where painful sensations are not present and how much of the body is not in pain	Large group inquiry and small group sharing about pleasant events in daily life during the week. Partners are also taught the pain practices in order to let them understand from their own experience how to offer labor support with calm and ease	Continue Awareness of Breathing practice 5–10 min every day Alternate yoga with the Body Scan Participants are encouraged to do at least one sequence of pain practices for 20–30 min, alternating 1-min holding ice using a variety of options with 1.5 min of Awareness of Breathing between the “ice contractions”	Continue Being with Baby practice Continue Mindfulness of Routine Activities, bringing awareness directly to any stress reactions (the contractions of life) experienced during the week Informal Pain Practice: Bring attention to any physical discomforts such as back pain, sciatica, shortness of breath, or heartburn. Practice being with whatever sensations are present, even if they are unpleasant or challenging Unpleasant Events Calendar
5	During the ice practice, both partners receive instruction for using mindful, compassionate touch and also experiment with various postures that might be used during labor, such as child’s pose, side lying, gently rocking side to side or back and forth while standing or sitting These practices may be very beneficial for partners for supporting pregnant women during childbirth contractions	Sitting Meditation, including sound as an object of awareness Mindful Yoga 3 Step Breathing Space Pain Practice 2: Working with partners using compassionate touch	Small and large group inquiry about participants’ experiences with mindfulness and compassion practices and the observations regarding pleasant and unpleasant experiences from the home practice of keeping the pleasant and unpleasant events calendars. Exploration of topics such as reacting or responding, desire and attachment, aversion and resistance and their relationship to secondary suffering in childbirth, parenting, and any experience in life	Continue Awareness of Breathing practice 5–10 min every day Continue to alternate yoga with the Body Scan Participants are encouraged to do at least one sequence of pain practices for 20–30 min, adding compassionate touch practices with their partner, making sure each of them has a turn being touched and touching and discovering what is useful for them	Continue Being with Baby practice. Continue Mindfulness of Routine Activities. Continue informal pain practice when physical discomforts arise in everyday life. Mindful Pooping for both expectant parents. 3 Step Breathing Space several times each day
6	Introduction to vocalizing low-pitched sounds as a way to focus attention and to work with intense body sensations during the ice practice Couples are encouraged to use all the previous practices they have learned in classes 4 and 5 to cope with the intense sensations of “ice contractions”	Sitting Meditation, including thoughts, emotions and choiceless awareness Mindful Yoga 3 Step breathing space Pain Practice 3: Full Immersion in very cold water	Teaching about causes and conditions and using mindful awareness for making wise choices in childbirth such as selection of a care provider and place of delivery Participants learn that the future is unknown, and there is no one “correct way” to give birth and that with continued practice they will have a variety of skills to work with pain and whatever may come during the birth process	Alternate sitting meditation with either the Body Scan or yoga One sequence of pain practices for 20–30 min integrating all the tools learned in classes 4, 5, and 6	Continue Awareness of Breathing practice 5–10 min every day and/or 3 Step Breathing Space Continue Being with Baby practice Continue Mindfulness of Routine Activities, including mindful pooping Continue informal pain practice when physical discomforts arise in daily life
7	Participants learn how mindfulness can help them cope with the biological, emotional, and social needs of a newborn and challenges that may appear during the postpartum period. Fears about the future and how to foster happiness and wellbeing in one’s self and one’s partners are addressed in an interpersonal mindful speaking and listening inquiry. A practice of self-compassion is introduced for the first time here	Sitting meditation Mindful Yoga 3 Step Breathing Space Loving-kindness meditation (including baby, oneself, partner, loved ones, all babies, and parents in the room, a neutral person, a difficult person, and finally all beings everywhere) Walking meditation Mindful speaking and listening inquiry Self-compassion meditation	Large group inquiry exploring participants’ experiences of Loving-kindness and self-compassion practices Interpersonal mindful speaking and listening inquiry between partners. This practice promotes an experience of feeling the authentic presence of the other person, of being seen and heard, as a valuable skill for parenting and partnership	Sitting Meditation with audio recordings. Alternate with either the Body Scan or yoga One sequence of pain practices for 20–30 min, integrating all the tools learned in classes 4, 5, and 6	Continue Awareness of Breathing practice 5–10 min every day. Continue Being with Baby practice. Experiment with adding phrases of Loving-kindness to this practice. Continue Mindfulness of Routine Activities, including mindful pooping. Continue informal pain practice when physical discomforts arise in everyday life Participants are encouraged to begin bringing attention to how they will find support for themselves as a newly birthed family during the early postpartum period 3 Step Breathing Space: several times throughout the day, particularly when feeling stress, as a preparation for postpartum there will probably be less time for formal meditation practice
8	Participants learn how the continued practice of mindfulness and compassion skills during postpartum may - promote resonance, attachment and bonding with the baby; - support the normal physiology of breastfeeding; - help to alleviate the stress of sleep deprivation and meeting the needs of a newborn Symptoms of postpartum blues and depression in both women and men are reviewed, including how to obtain help from a health provider if necessary. Course review and closing ceremony	Sitting Meditation, including thoughts, emotions and choiceless awareness Mindful yoga 3-Step Breathing Space Loving-kindness final meditation	Reflections on what participants have learned throughout the program and final sharing with mindful listening and speaking Participants are encouraged to continue their practice and, if possible, to continue to meet after the formal course ends, forming a community of support for sustaining the practice of living and parenting mindfully	Participants are invited to practice meditation for 30 min each day without the audio recordings in the coming weeks They are also encouraged to continue to incorporate Loving-kindness and self-compassion into their formal practice and daily life	Continue Awareness of Breathing 5–10 min every day Continue Being with Baby practice. Experiment with adding phrases of Loving-kindness to this practice Continue Mindfulness of Routine Activities, including mindful pooping Continue informal pain practice when physical discomforts arise in everyday life Pay attention to bringing mindfulness into communication with others (mindful speech) Continue practicing the 3-Step Breathing Space
9	The class reunion that takes place 3 months after birth is an opportunity for the new parents to reconnect, to meet each other’s babies, and to reflect on what they learned from their birth experience Participants are invited to share how they are applying mindfulness and compassion skills in parenting, how they are growing together as new parents and to express appreciation to and about their partner and themselves Postpartum blues and depression and challenges in breastfeeding are also addressed, including how to seek help from a health provider if necessary	10-min practice of sitting meditation with babies 10-min practice of loving kindness meditation and self-compassion meditation with babies	Large group sharing about how they applied mindfulness and compassion practices to their experiences of birth and how they are using these practices to help them cope with postpartum challenges. Participants are again encouraged to continue to meet to support their ongoing use of mindfulness and compassion practice as a foundation for living and parenting mindfully	3-Step Breathing Space several times a day when possible Self-compassion and loving-kindness practice when possible	Continue informal practice while breastfeeding, taking care of their baby, and doing daily activities Participants are encouraged to do some formal practice or return to formal practice as their babies grow
10	This class takes place as a reunion 6 months after giving birth. Participants share how to apply mindfulness and compassion skills to - the challenges of postpartum and breastfeeding; - going back to work; - finding childcare; - adapting to their new roles, including the changes in their relationship as a couple and with their families of origin	10-min practice of sitting meditation with babies 10-min practice of Loving-kindness meditation and self-compassion meditation with babies	Large group sharing about their experiences of parenting and how they are using mindfulness and compassion practices creatively in this period of their lives	3-Step Breathing Space several times a day when possible Self-compassion and loving-kindness practice when possible	Continue informal practice while breastfeeding, taking care of the baby, at work and doing daily activities Participants are encouraged to do some formal practice or return to formal practice as their babies grow
